# Effect of Alzheimer's Disease Risk Variant rs3824968 at *SORL1* on Regional Gray Matter Volume and Age-Related Interaction in Adult Lifespan

**DOI:** 10.1038/srep23362

**Published:** 2016-03-21

**Authors:** Chu-Chung Huang, Mu-En Liu, Hung-Wen Kao, Kun-Hsien Chou, Albert C. Yang, Ying-Hsiu Wang, Tong-Ru Chen, Shih-Jen Tsai, Ching-Po Lin

**Affiliations:** 1 Institute of Neuroscience, National Yang-Ming University, Taipei, Taiwan; 2 Department of Psychiatry, Taipei Veterans General Hospital, Taipei, Taiwan; 3 Institute of Brain Science, National Yang-Ming University, Taipei, Taiwan; 4 School of Medicine, National Yang-Ming University, Taipei, Taiwan; 5 Department of Radiology, Tri-Service General Hospital, National Defense Medical Center, Taipei, Taiwan; 6 Brain Research Center, National Yang-Ming University, Taipei, Taiwan; 7 Center for Dynamical Biomarkers and Translational Medicine, National Central University, Chungli, Taiwan; 8 Department of Biomedical Imaging and Radiological Sciences, National Yang-Ming University, Taipei, Taiwan

## Abstract

Sortilin receptor 1 (*SORL1*) is involved in cellular trafficking of amyloid precursor protein and plays an essential role in amyloid-beta peptide generation in Alzheimer disease (AD). The major A allele in a *SORL1* single nucleotide polymorphism (SNP), rs3824968, is associated with an increased AD risk. However, the role of *SORL1* rs3824968 in the normal ageing process has rarely been examined in relation to brain structural morphology. This study investigated the association between *SORL1* rs3824968 and grey matter (GM) volume in a nondemented Chinese population of 318 adults within a wide age range (21–92 years). Through voxel-based morphometry, we found that participants carrying *SORL1* allele A exhibited significantly smaller GM volumes in the right posterior cingulate, left middle occipital, medial frontal, and superior temporal gyri. Considerable interaction between age and *SORL1* suggested a detrimental and accelerated ageing effect of allele A on putamen. These findings provide evidence that *SORL1* rs3824968 modulates regional GM volume and is associated with brain trajectory during the adult lifespan.

The sortilin-related receptor gene (*SORL1*) encodes a mosaic protein belonging to at least two families: the vacuolar protein sorting 10 domain-containing receptor family and the low density lipoprotein receptor family. The encoded protein acts as an apolipoprotein E (APOE) binder and sorting receptor that regulates the intracellular transport and processing of the amyloid precursor protein (APP) in neurons^1^. In addition to the ε4 allele of APOE, the major genetic risk factor for late-onset Alzheimer disease (AD)^2^, *SORL1* is genetically associated with late-onset and sporadic AD^3^. *SORL1* regulates and traps APP in the Golgi apparatus and reduces the production of the amyloid-beta peptide (Aβ), the main component of senile plaques in AD^4^. Cell biology experiments have indicated that *SORL1* underexperssion leads to overproduction of Aβ^3^, whereas *SORL1* overexpression considerably reduces cellular APP and extracellular Aβ^5^. Although the underlying mechanisms of *SORL1* in AD and neurodegenerative processes remain unclear, a recent comprehensive review on multiple functional domains of *SORL1* suggested that *SORL1* can assume various characters in AD pathogenesis^6^.

Among single nucleotide polymorphisms (SNPs) in *SORL1*, Rogaeva *et al.*^3^ observed a substantial association between a synonymous SNP in exon 34 (rs3824968) and AD in a northern European case–control cohort and implied that the SNP alters the physiological role of *SORL1* in the processing of APP holoprotein. The association between *SORL1* rs3824968, neurodegenerative disease, and ageing process has recently been reported^6^,^7^,^8^,^9^,^10^,^11^. Lower levels of CSF Aβ_1-42_ were observed in carriers of the AD risk allele A in *SORL1* rs3824968 compared with noncarriers^12^, and the decrease in these levels was associated with AD progression^13^. Two studies in the Chinese population^9^,^14^ also reported a similar association between *SORL1* rs3824968 A allele and increased AD risk. However, the association between *SORL1* rs3824968 and AD was not consistent across different populations. This discrepancy may be owing to ethnic difference, suggesting the existence of population-specific locus. It should be noted the reference allele for the *SORL1* rs3824968 is the T over all populations. In the Chinese population, however, the reference allele is the A. The association between *SORL1* variants and accelerating cognitive ageing in relation to spatial and episodic memory performance were observed in a follow-up study of a nondemented population^15^. In the Lothian Birth Cohort comprising 1936 participants, an association between the *SORL1* rs3824968 A allele and reduced spatial-span tasking in cognitively healthy participants was noted^16^. Bralten *et al.*^17^ observed associations between *SORL1* variants and hippocampal volume, as an intermediate phenotype, in a gene-wide association analysis of healthy young adults.

In addition to the differences in the effects of the SNP alleles within the brain, the expression of *SORL1* can vary according to the age grades during the lifespan. A previous study reported that healthy elderly individuals exhibit two-fold higher *SORL1* expressions than do younger adults^18^. Notably, *SORL1* risk variants may predict decrease in white matter (WM) integrity and *SORL1* mRNA expression, most prominently during the ageing process, and they may be associated with high Aβ deposition in the postmortem brain, thus conferring neuropathological risk for *SORL1* variants through the amyloidogenic pathway^11^. Nonetheless, few studies have systemically investigated the interactions of age, *SORL1* effect, and regional grey matter (GM) volume changes in a healthy population. Thus, deducing the risk factors for accelerated brain ageing by evaluating the interaction of *SORL1* variants with age and their association with the regional brain volume changes over the adult lifespan would benefit current ageing-related genetic imaging studies.

Considering previous findings and the role of *SORL1* in the normal ageing process, this study examined regional GM volume changes as an intermediate phenotype in nondemented adults during their lifespan to infer the relationship between the genetic effect of *SORL1* rs3824968 and brain ageing. By using a relatively large sample size with a broad age range in combination with an unbiased and whole-brain voxel-based morphometry (VBM) approach, we assessed two hypotheses based on previous studies: (1) independent of the age effect, the variants of *SORL1* rs3824968 are associated with the difference of regional GM volume in nondemented adults and (2) *SORL1* rs3824968 interacts with age and is associated with regional GM volume alterations across lifespan.

## Results

### Demographics

Twelve participants were excluded because of failure in *SORL1* genotyping or extensive motion problems during MRI scanning. Of the 318 participants that completed *SORL1* genotyping, MRI acquisitions, and cognitive assessments, 42, 146, and 130 carried the *SORL1* TT, AT, and AA genotypes, respectively. APOE genotypes were also determined to reveal five genotypes: ε2/ε2 (*n* = 3, 0.9%), ε2/ε3 (*n* = 44, 13.8%), ε3/ε3 (*n* = 222, 69.8%), ε2/ε4 (*n* = 6, 1.9%), and ε3/ε4 (*n* = 42, 13.2%). The frequencies of the genotypes followed the Hardy–Weinberg equilibrium. The demographics and clinical characteristics of the study group are shown in Table 1. The participants carrying the *SORL1* TT, AT, and AA genotypes showed no significant differences in age (*P* = 0.230), sex (*P* = 0.695), educational level (*P* = 0.380), APOE allele distribution (*P* = 0.291), total GM volume (*P* = 0.304), total WM volume (*P* = 0.243), total CSF volume (*P* = 0.849), total intracranial volume (*P* = 0.425), or neuropsychological characteristics.

### Main Effect of Age and *SORL1* rs3824968 on Regional GM Volume

**Figure 1:**
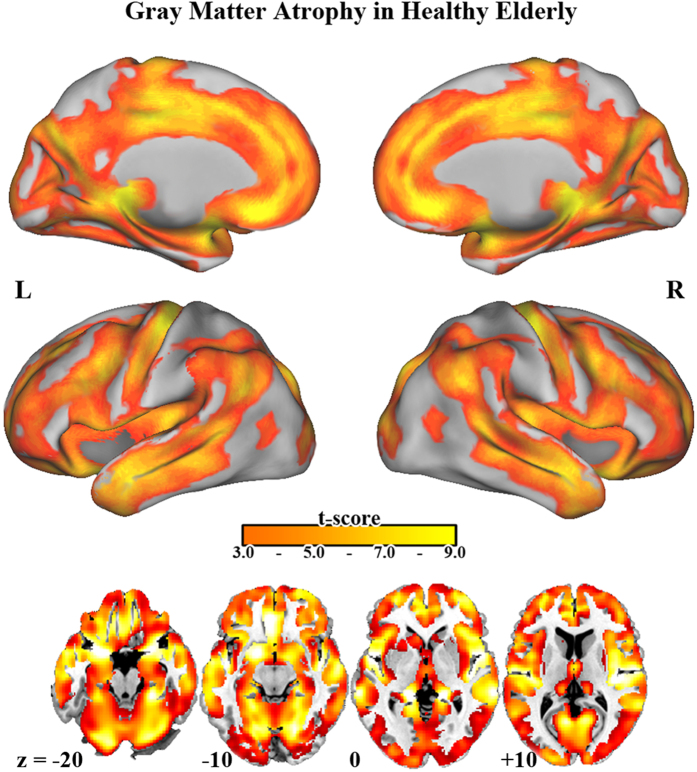
Age effect: GM differences in a nondemented elderly participants. Regions of significant GM differences from 318 normal participants superimposed on surface space. T-score maps show clusters at a voxel threshold with *P* of <0.005 as well as extended voxel sizes of 298, all of the clusters remained significant and survived from the criteria of corrected *P*_alpha_ of <0.05 by Monte Carlo simulation.

**Figure 2:**
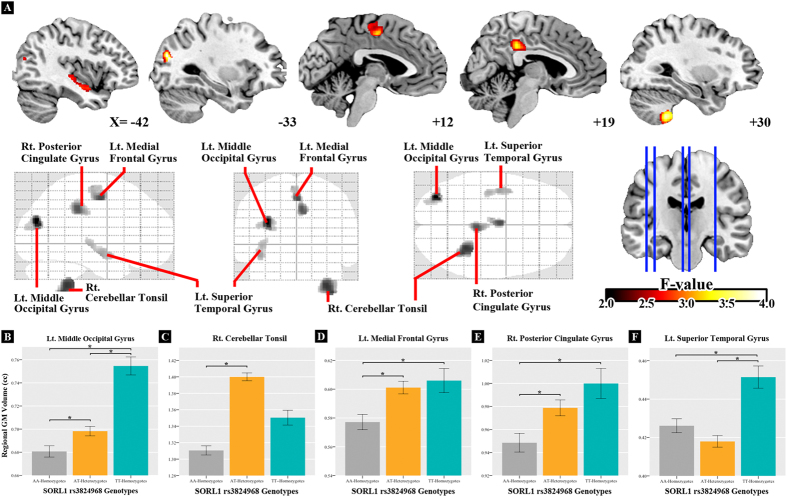
Regional GM volume differences among the three *SORL1* rs3824968 genotypic groups. T-score map shows significant smaller GM volume in *SORL1* A allele carriers compared with those carrying TT (**A**). The clusters were set at a voxel threshold with *P* of <0.005 as well as extended voxel sizes of 298, all of the clusters remained significant and survived from the criteria of corrected *P*_alpha_ of <0.05 by Monte Carlo simulation. Bottom bar graph shows the GM volume difference between the *SORL1* genotypes and the regions with significant gene main effect in the left middle occipital gyrus (**B**), right cerebellum tonsil (**C**), left medial frontal gyrus (**D**), right posterior cingulate gyrus (**E**), and left superior temporal gyrus (**F**). ^*^Bonferroni-corrected *P* < 0.05 (post hoc tests in analysis of covariance).

Regarding the main effect of age, DARTEL-based T1 VBM analyses showed widespread age-related GM volume differences in the study participants (Fig. 1), in accordance with previous studies^19^,^20^. Regarding the main effect of the gene obtained from F tests (Table 2, Fig. 2A), on comparing the participants carrying the *SORL1* TT, AT, and AA genotypes, the post hoc analyses revealed positive dosage effect of the T allele on GM volume of the left middle occipital gyrus (Fig. 2B). In addition, participants carrying AA showed lower GM volume in the right cerebellar tonsil than did those carrying AT (Fig. 2C). Moreover, participants carrying AA had lower GM volumes in the left medial frontal and right posterior cingulate gyri than did those carrying the T allele (Fig. 2D,E). The participants carrying the A allele showed significantly lower GM volume in the left superior temporal gyrus than did those carrying TT (Fig. 2F). After controlling APOE genotype as additional nuisance variable, the findings remain consistent with current findings (Supplementary Table 1).

### Age and *SORL1* Interaction Effect on Regional GM Volume

**Figure 3:**
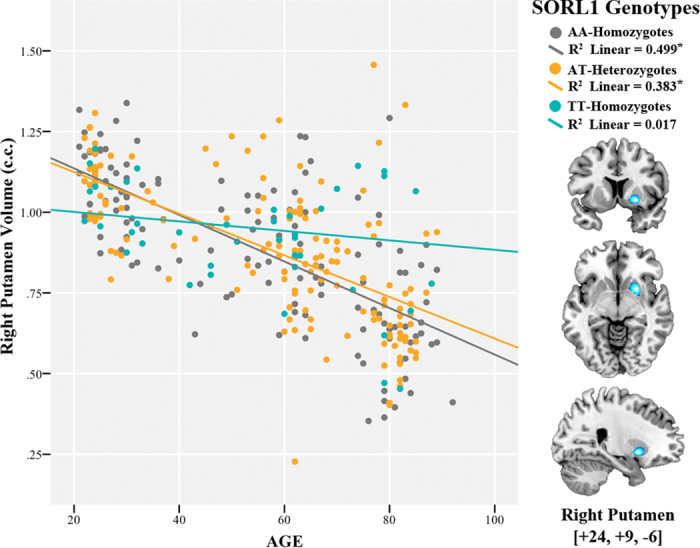
Interaction between the *SORL1* genotype and age on right putamen GM volume. The scatter plot demonstrates the interaction between the *SORL1* genotype and age on right putamen GM volume using voxel-wised covariate analysis with the *SORL1* genotypes as the condition and age as the covariate, while controlling for sex and education level as nuisance variables (corrected *P*_alpha_ of <0.05 by Monte Carlo simulation)).

Regarding the voxelwise covariate interaction analysis, we observed considerable interaction effect between age and the three *SORL1* genotypes in the right putamen region (Fig. 3), and the downward slope was steeper in the participants carrying AA and AT than in those carrying TT. To verify whether this accelerated reduction in the putaminal volume was related to cognitive performance, we applied partial correlation for each genotypic group (results are presented in *Section 3.4*).

### Correlation Analysis Between Regional GM Volume and Cognitive Function

In the partial correlation analysis between regional GM volume and cognitive performance levels in all participants, none of the regional GM volume changes for the effect of *SORL1* showed correlation with MMSE, digit span forward (DSF), or digit span backward (DSB) scores. For the correlation between the cluster of voxelwise interaction and cognitive tests, although the results failed to survive from the criteria of Bonferroni correction, a positive trend between DSB score and putaminal volume was observed in the participants carrying AA (r = 0.189, *P* = 0.034).

## Discussion

We performed VBM analysis to examine the effect of *SORL1* rs3824968 on regional GM volume and age-related changes in the brain GM volume of nondemented participants within a wide age range. The findings of this study showed that the *SORL1* rs3824968 A allele carriers had lower GM volume in the left superior temporal, left medial frontal, right posterior cingulate, and left middle occipital gyri than did those carrying TT. A considerable interaction between age and the *SORL1* genotypes was observed, showing an accelerated (downward) slope of GM volume differences in the right putamen of the participants carrying the AD-risk (A) allele compared with those carrying TT. Thus, our findings support the hypothesis that *SORL1* rs3824968 is associated with regional GM volume differences in early adulthood and can influence ageing-related GM morphological changes during the nondemented adult lifespan.

The *SORL1* rs3824968 A carriers showed significantly decreased GM volume in the left superior temporal, left medial frontal, right posterior cingulate, and left middle occipital gyrus, suggesting detrimental effects of the A allele on brain GM volume. A trend towards gene dosage effect of the A allele was also observed in the left middle occipital, medial frontal, and right posterior cingulate gyri. *SORL1* SNPs have been found to be associated with an increased risk of neurodegenerative disease in Swedish and African American population through meta-analysis^21^,^22^. Nonetheless, association studies have further identified that haplotype SNPs including rs3824968 are associated with lower Aβ_42_ levels in CSF and higher AD risk in Asian population^14^,^23^,^24^. Therefore, the *SORL1* risk variants may alter the Aβ expression levels in either healthy adults or patients with neurodegenerative disease^12^,^13^,^25^, thus suggesting that *SORL1* rs3824968 variants may influence amyloid pathology during ageing. Greater amyloid burden has been shown to be positively associated with regional GM atrophy^26^,^27^,^28^, particularly in the superior temporal, medial frontal, and cingulate gyri in both structural and functional MRI studies^29^,^30^. In addition to the hypothesis of amyloid pathology, Cuenco *et al.*^31^ proposed another viewpoint that *SORL1* increases dementia risk through effects on cerebrovascular abnormalities; *SORL1* rs3824968 and its haplotypes were found to be associated with brain volume and WM hyperintensities in their study. This biological and neuroimaging evidence may suggest that *SORL1* rs3824968 is associated with brain morphology in terms of A allele-related volumetric reduction caused by high Aβ load, cardiovascular disease, or both. However, additional studies are necessary to explore the potential mechanisms of *SORL1* rs3824968 on the cellular and molecular determinants of brain structural differences in both demented and nondemented individuals.

Here, we revealed the first evidence of the influence of *SORL1* on age-related changes in the brain cortical morphology of nondemented participants over a wide age range. The right putamen of the participants carrying AA and AT showed a considerable effect of interaction between age and the *SORL1* genotype with a steeper downward slope of age-related GM volume reduction compared with that of those carrying TT. This finding supports the hypothesis that *SORL1* polymorphism plays a role in the ageing process of the human brain with a partially detrimental effect on age-related differences of regional GM volume, particularly in the putamen of the participants with the A allelic variant. A previous study reported that participants with presenilin-1, presenilin-2, or APP mutations showed higher amyloid load and greater GM atrophy in the putamen^32^. In other words, if the *SORL1* variants contribute to the risk of AD neuropathogenesis through the amyloidogenic pathway, putaminal volume could be a vulnerable subcortical area in the brain ageing process with regard to the *SORL1* effect. In addition, such an accelerated regional GM volume reduction effect in the putamen further links to cognitive decline in participants carrying the A allele. A trend of positive correlation between putamen volume and DSB scores was observed in the participants carrying AA. Studies have shown that the basal ganglia play a vital role in working memory and executive function, particularly in the putaminal region^33^,^34^. Moreover, increased activity in the putamen during a card sorting test was observed to be related to working memory function, as evident on functional MRI^35^. In the current study, imaging indicated an association between *SORL1* rs3824968 and putaminal volume and a trend of neural correlation of working memory (DSB), which may be a marker of accelerated cognitive impairment at preclinical stage^36^,^37^,^38^.

We observed no age-by-SNP interaction effect in the significant clusters that detected the main effect of genotype: the left superior temporal gyrus, left middle occipital gyrus, left medial frontal gyrus, right cerebellar tonsil, and right posterior cingulate gyrus. In addition to a previous study, which proposed a pathway by which *SORL1* variants could mediated neural risk of AD beginning from teenage years^25^, current data support two pathways of *SORL1* rs3824968 effect on GM volume during the ageing process: (1) differences of regional cortical volume between the three genotypes occur from early adult life and preferentially stay stable during adulthood and (2) in contrast to the first pathway, variants of *SORL1* rs3824968 do not modulate putaminal volume directly but interact with age during the adult lifespan. Further research is necessary to explore underlying mechanisms for various *SORL1* genetic effects along brain ageing trajectory.

The necessity for statistically sufficient sample sizes in imaging studies of genetic variation is increasingly being recognised. The relatively large and (by international standards) homogenous cohort of participants with a wide age range recruited in this study increased the credibility of our findings, based on previously proposed recommendations regarding cohort sizes^39^. However, the characteristic cross-sectional design of this study may be a limitation. We did not measure Aβ load levels in our participants; the genotypic effect of the *SORL1* SNP on GM volume might be affected through Aβ deposition or other mechanisms such as inflammatory signalling modulation^40^. Therefore, the associations between *SORL1* variants, biochemical data, and brain morphometry should be investigated simultaneously in future studies to explain the effect of *SORL1* on GM volume reduction. In addition, rather than having a direct effect on regional GM volume, *SORL1* rs3824968 may be in linkage disequilibrium with truly associated variants; in addition, it may be an intermediate phenotype. Since such a linkage could vary among different populations and the association between rs3824968 and AD was not replicated in studies of other populations, these may confound the generalisability of our findings, which were based on a homogenous Chinese cohort. In addition, no significant correlation was found between general cognitive function (MMSE, DSF, and DSB) and regional GM volume differences of the *SORL1* effect. Although MMSE is a widely used tool for detecting dementia, it may lack sensitivity to and specificity for early signs of subtle cognitive changes. Despite the current samples being from nondemented participants, the ceiling effect of using MMSE, DSF, and DSB for cognition assessment may also have yielded false negative results^41^. Specific cognitive assessments regarding short- and long-term memory should be examined in future to associate cognitive decline with genetic effect and brain structural changes.

The AD-risk *SORL1* rs3824968 A allele was associated with decreased GM volume and exhibited a trend towards gene dosage effect in several brain regions during the adult lifespan. Considerable interaction between age and *SORL1* suggested a detrimental and accelerated ageing effect of the AD-risk allele on putaminal volume. Although the underlying molecular mechanisms remain unclear, our findings support the hypothesis that *SORL1*-related genetic factors play a vital role in the process of normal ageing. The regional GM volume alterations associated with the effect of *SORL1* rs3824968 might be a potential neuroimaging biomarker for accelerated brain ageing during the adult lifespan.

## Methods

### Participants and Instruments

This study initially recruited 330 nondemented ethnic Chinese participants from Northern Taiwan (mean age: 55.9 ± 22.1 years; range: 21–92 years; 57.5% males) through an advertisement in local communities and universities. Each participant was administered a diagnostic structured Mini-International Neuropsychiatric Interview (MINI)^42^, Mini-Mental State Examination (MMSE) Chinese version^43^, and Clinical Dementia Rating (CDR) scale^44^. Participants with any of the following conditions were excluded: (1) a Diagnostic and Statistical Manual of Mental Disorders, Fourth Edition (DSM-IV) axis I diagnosis such as mood disorder or psychotic disorder; (2) a neurobiological disorder, such as dementia, head injury, stroke, or Parkinson disease; (3) illiteracy; (4) an MMSE score of ≤24; or (5) ≥65 years of age with a CDR of >0.5 [exclusion criteria (4) and (5) were applied to prevent possible inclusion of dementia patients].

The cognitive functioning of the participants was evaluated using the MMSE for general cognitive status and the Wechsler Digit Span subtest for verbal working memory abilities. All participants exhibited sufficient visual and auditory acuity to undergo cognitive testing. This study was conducted in accordance with the Declaration of Helsinki and approved by the Institutional Review Board of Taipei Veterans General Hospital. Written informed consent was obtained from all participants after they were adequately apprised of the study.

### Genotyping

Genomic DNA was extracted from peripheral blood with a commercial kit (Qiagen, Gentra Puregene Blood Kit). *SORL1* rs3824968 genotyping was performed using high-throughput matrix-assisted laser desorption/ionisation time-of-flight mass spectrometry. PCR and single-base extension primers were designed using DNA mass arrays (Sequenom, San Diego). The genotyping analysis was performed using an iPLEX Gold Reagent Kit according to the manufacturer’s instructions. The purified extension products were spotted onto a 384 SpectroCHIP II array by using a MassArray Nanodispenser RS1000, followed by an analysis on a MassARRAY Compact Analyzer. The resulting spectra were processed, and the alleles were called using a SpectroTYPER. The APOE genotype was determined using the PCR-restriction fragment length polymorphism method, as described by Hong *et al.*^30^.

### MRI Acquisition

All MRI scans were performed at National Yang-Ming University, Taipei, Taiwan, on a 3.0-T Siemens MRI scanner with a 12-channel head coil (Siemens Magnetom Tim Trio, Erlangen, Germany). High-resolution structural MR images were acquired through sagittal 3D magnetisation-prepared rapid gradient-echo sequencing (TR = 2530 ms, TE = 3.5 ms, TI = 1100 ms, FOV = 256 mm, flip angle = 7°, matrix size = 256 × 256, 192 sagittal slices, voxel size = 1.0 × 1.0 × 1.0 mm, no gap). All images were acquired parallel to the anterior commissure–posterior commissure line. To minimise motion artefacts generated during image acquisition, each participant’s head was immobilised using cushions inside the coil. An experienced radiologist carefully checked each image to ensure the absence of scanner artefacts, motion problems, or gross anatomical abnormalities.

### Diffeomorphic Anatomical Registration Through Exponentiated Lie Algebra-Based T1 VBM Analysis

Individual T1-weighted volumetric images were processed using Gaser’s VBM8 toolbox (http://dbm.neuro.uni-jena.de) within Statistical Parametric Mapping (SPM8; Wellcome Institute of Neurology, University College London, UK). VBM processing was performed using the following procedures: (1) The anterior commissure was set as the origin of each T1-weighted image. (2) A segmentation approach in the VBM8 toolbox was applied in the initial native space. (3) To achieve a higher registration accuracy and account for different brain size among subjects, the native space segments of the GM, WM, and cerebrospinal fluid (CSF) were initially affine registered to the tissue probability maps in the Montreal Neurological Institute (MNI) standard space. (4) All affine registered GM and WM tissue segments were used to generate group-specific templates through nonlinear warping by using the Diffeomorphic Anatomical Registration Through Exponentiated Lie (DARTEL) algebra toolbox^45^ implemented in SPM8. (5) Nonlinear deformation parameters obtained in the previous step were used to modulate the GM, WM, and CSF tissue maps of the participants’ brains to compare actual volumetric differences among groups. (6) Modulated tissue segments were converted into an isotropic voxel resolution of 1.5 × 1.5 × 1.5 mm. All normalised, segmented, and modulated MNI standard space images were then smoothed with an 8-mm Gaussian kernel before voxelwise group comparisons. Segmented tissue volumes ((i.e. (GM, WM, and CSF) were estimated in cubic millimetres by counting the voxels representing the native space of the GM, WM, and CSF. Total intracranial volume was calculated as the sum of the GM, WM, and CSF volumes.

### Statistical Analysis

Statistical analysis was performed using the Statistical Package for Social Sciences (SPSS) software package (SPSS 20 for Windows, Chicago, IL, USA). Analysis of variance and chi-squared test were respectively applied to compare the continuous and categorical variables of the demographic data among the three groups (participants with the AA, AT, and TT genotypes). Smoothed and modulated GM segments were analysed with SPM8 by using the framework of a general linear model. Analysis of covariance was used by covarying age, sex, and educational levels to reveal the random effect of *SORL1* rs3824968 on GM volumes. To prevent possible partial volume effects around the margin between the GM and WM, all voxels with a GM probability value of <0.2 (range: 0–1) were eliminated. In this study, to further investigate the main effects of age-by-SNP interaction on regional GM volumes, voxelwise covariate interaction analysis was used with the *SORL1* genotype as a condition and age as a covariate, controlling for sex and education level as nuisance variables. In each model, the main effect and interaction were explored using F tests. Post hoc *t* tests were subsequentially performed to examine the relationships between genotypic groups for any significant main effects and interaction clusters using Bonferroni corrections for multiple comparisons. The problem of multiple comparisons was corrected using a Monte Carlo simulation created in AlphaSim and implemented in the Analysis of Functional NeuroImages software (http://afni.nimh.nih.gov/afni/). Based on the simulation, the statistical threshold of F tests and post hoc tests were set at corrected *P*_alpha_ of <0.05, with a minimum cluster size of 298 voxels (AlphaSim with the following parameters: single voxel *P* value of 0.005, 5000 simulations, FWHM_x/y/z_ = 7.5/8.7/8.1 mm with a GM mask) for multiple comparisons. The coordinates of each significant cluster were transformed from MNI coordinates into Talairach coordinates by using the GingerALE toolbox (BrainMap Development Team; http://brainmap.org/ale/index.html). The anatomical structures of the coordinates representing significant clusters were identified using the Talairach and Tournoux atlas^46^. The volume of each significant cluster was extracted from modulated GM segments in MNI space for each participant and further correlated to cognitive assessments in the entire study group. Partial correlation analyses between regional GM volume (crucial results of genetic effect/age-by-SNP interaction) and cognitive performance were controlled for nuisance variables (age, sex, and educational levels) and corrected for multiple testing with Bonferroni correction. Since APOE is the major risk factor for AD, additional statistical analyses were performed using age, sex, educational level, and APOE genotype (ε2/ε2, ε2/ε3, ε3/ε3, ε2/ε4, and ε3/ε4) as nuisance variable to account for potential effect by APOE status.

## Additional Information

**How to cite this article**: Huang, C.-C. *et al.* Effect of Alzheimer’s Disease Risk Variant rs3824968 at *SORL1* on Regional Gray Matter Volume and Age-Related Interaction in Adult Lifespan. *Sci. Rep.* **6**, 23362; doi: 10.1038/srep23362 (2016).

## Supplementary Material

Supplementary Information

## Figures and Tables

**Table 1:** Demographic and clinical characteristics among *SORL1* rs3824968 genotypic groups.

Variables	AA- Homozygotes	AT- Heterozygotes	TT- Homozygotes	*P*-Value
	N = 130	N = 146	N = 42	
Age (years)	53.9 (22.7)	58.2 (21.6)	53.9 (21.5)	0.230
Education (years)	12.6 (6.7)	12.2 (5.9)	13.7 (5.4)	0.380
Women*	56 (43.1)	59 (40.4)	20 (45.5)	0.695
Handedness (R/L)	126/4	142/4	42/0	0.527
APOE-ε4 carriers*	24 (18.6)	20 (13.7)	4 (9.5)	0.291
Digits Span Forward	13.8 (2.6)	13.3 (2.8)	13.8 (2.2)	0.296
Digits Span Backward	7.9 (4.3)	7.1 (3.8)	7.8 (3.9)	0.174
MMSE Score	27.9 (2.4)	27.7 (2.5)	28.33 (1.5)	0.330
GM Volume (liter)	0.602 (0.065)	0.599 (0.066)	0.617 (0.068)	0.304
WM Volume (liter)	0.504 (0.061)	0.495 (0.052)	0.509 (0.065)	0.243
TIV (liter)	1.379 (0.119)	1.368 (0.113)	1.394 (0.134)	0.425

Data are presented as mean (standard deviation) and ^*^number (percentage). *P* values were derived from analysis of variance. Abbreviations: MMSE, Minimental State Examination; GM, grey matter; WM, white matter; TIV, total intracranial volume; APOE, apolipoprotein E.

**Table 2:** Regional GM volume differences among the three *SORL1* rs3824968 genotypic groups.

MNI Coordinates			Voxel size	Brain region	Regional GM Volume Mean (SE) (cm^3^)			F-Value
x	y	z			AA- Homozygotes	AT- Heterozygotes	TT- Homozygotes	
−33	−79	22	378	Left Middle Occipital Gyrus (BA 19)	0.677 (0.008)	0.703 (0.007)	0.748 (0.013)	11.52
30	−46	−51	746	Right Cerebellum Tonsil	1.305 (0.017)	1.406 (0.017)	1.344 (0.031)	9.62
−2	−9	51	341	Left Medial Frontal Gyrus (BA 6)	0.572 (0.006)	0.607 (0.006)	0.600 (0.011)	9.03
3	−34	37	427	Right Posterior Cingulate Gyrus (BA 31)	0.941 (0.009)	0.988 (0.009)	0.992 (0.016)	8.72
−42	−9	−15	302	Left Superior Temporal Gyrus (BA 48)	0.423 (0.003)	0.421 (0.003)	0.446 (0.005)	6.90

Z-scores are for the peak statistical significant voxel of each regional cluster with corrected *P*_alpha_ of <0.05 (corrected for multiple comparisons by using Monte Carlo simulation) after controlling for age, sex, and education level. Abbreviations: BA, Brodmann area; GM, grey matter; MNI, Montreal Neurological Institute; SE, standard error.
