# Supplementary material for: Effect of Alzheimer's Disease Risk Variant rs3824968 at SORL1 on Regional Gray Matter Volume and Age-Related Interaction in Adult Lifespan
Source: Sci Rep. 2016 Mar 21;6:23362. doi: 10.1038/srep23362 (PMC4800313; doi:10.1038/srep23362)
Supplement: Supplementary Information [file srep23362-s1.doc]

**Effect of Alzheimer's Disease Risk Variant rs3824968 at *SORL1* on Regional Gray Matter Volume and Age-Related Interaction in Adult Lifespan**

Chu-Chung Huang^a,f^, Mu-En Liu^b,c,d^, Hung-Wen Kao^e,h^, Kun-Hsien Chou^a,f^, Albert C. Yang^b,d,g^, Ying-Hsiu Wang^b^, Tong-Ru Chen^b^, Shih-Jen Tsai^b,d*^, Ching-Po Lin^a,f,h*^

^a^Institute of Neuroscience, National Yang-Ming University, Taipei, Taiwan

^b^Department of Psychiatry, Taipei Veterans General Hospital, Taipei, Taiwan

^c^Institute of Brain Science, National Yang-Ming University, Taipei, Taiwan

^d^School of Medicine, National Yang-Ming University, Taipei, Taiwan

^e^Department of Radiology, Tri-Service General Hospital, National Defense Medical Center, Taipei, Taiwan

^f^Brain Research Center, National Yang-Ming University, Taipei, Taiwan

^g^Center for Dynamical Biomarkers and Translational Medicine, National Central University, Chungli, Taiwan

^h^Department of Biomedical Imaging and Radiological Sciences, National Yang-Ming University, Taipei, Taiwan

***Corresponding authors:**

Dr. Ching-Po Lin

E-mail: chingpolin@gmail.com

Institute of Neuroscience, National Yang-Ming University, 155, Li-Nong St. 112, Taipei, Taiwan

Tel: +886-2-2826 7338; Fax: +886-2-2826 2285

Dr. Shih-Jen Tsai

E-mail: tsai610913@gmail.com

Department of Psychiatry, Taipei Veterans General Hospital. No. 201, Shih-Pai Road, Sec. 2, 11217, Taipei, Taiwan

Tel: +886-2-2875 7027, ext. 276; Fax: +886-2-2872 5643

**Supplementary Table 1. Regional GM volume differences among the three *SORL1*** rs3824968 genotypic groups

| **MNI Coordinates** | | | **Voxel size** | **Brain region** | **F-Value** |
| --- | --- | --- | --- | --- | --- |
|
| **x** | **y** | **z** |
| -33 | -79 | 22 | 522 | Left Middle Occipital Gyrus (BA 19) | 12.06 |
| 30 | -46 | -51 | 729 | Right Cerebellum Tonsil | 9.55 |
| -2 | -9 | 51 | 351 | Left Medial Frontal Gyrus (BA 6) | 9.07 |
| 3 | -34 | 37 | 506 | Right Posterior Cingulate Gyrus (BA 31) | 9.08 |
| -42 | -9 | -15 | 339 | Left Superior Temporal Gyrus (BA 48) | 7.34 |

Z-scores are for the peak statistical significant voxel of each regional cluster with corrected *P*_alpha_ of <0.05 (corrected for multiple comparisons by using Monte Carlo simulation) after controlling for age, sex, education level, and APOE genotype.

Abbreviations: BA, Brodmann area; GM, grey matter; MNI, Montreal Neurological Institute; SE, standard error.

**Supplementary Figure 1. Interaction between the SORL1 genotype and age on right putamen GM volume (outlier removed)**


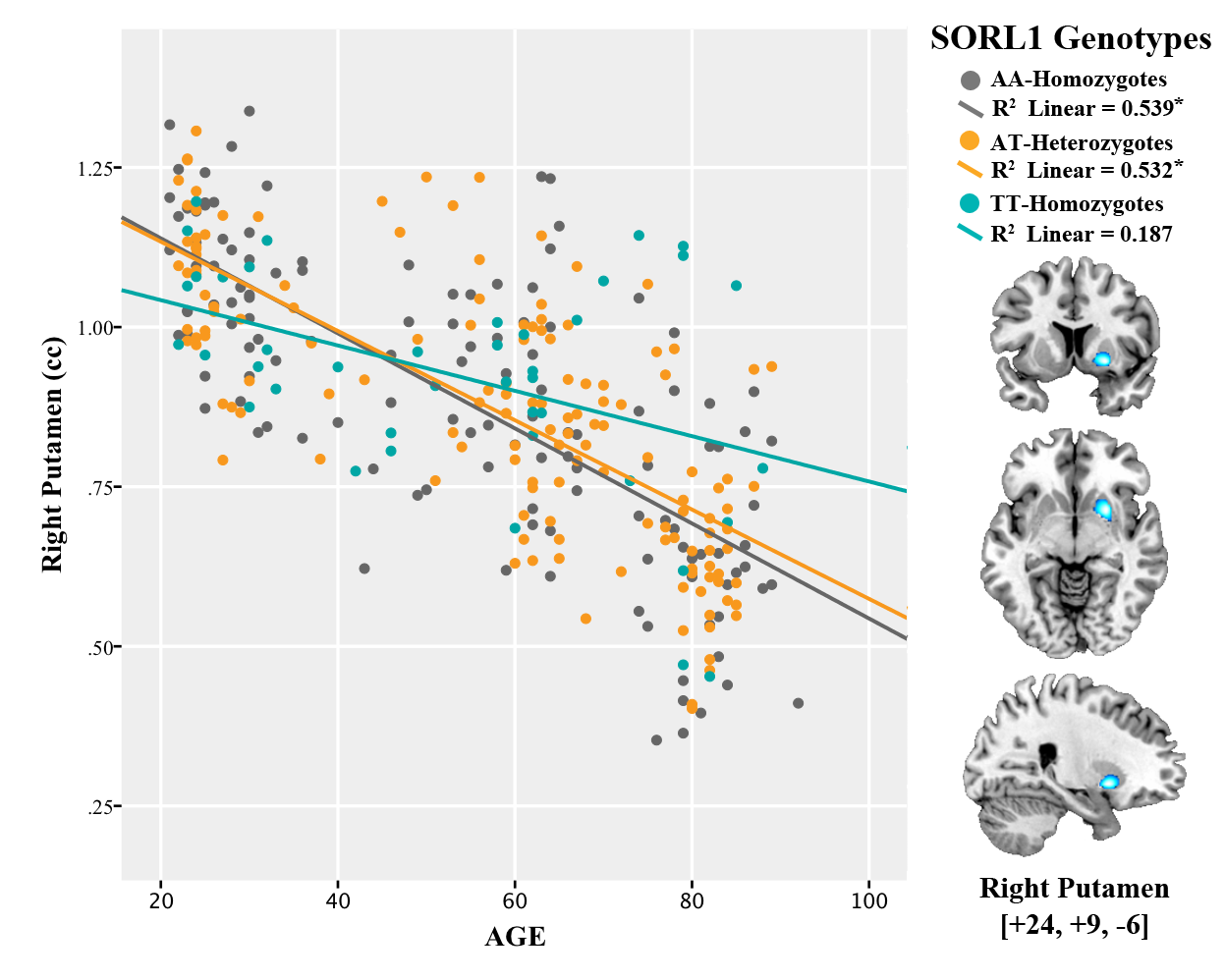


The scatter plot demonstrates the interaction between the *SORL1* genotype and age on right putamen GM volume using voxel-wised covariate analysis with the *SORL1* genotypes as the condition and age as the covariate, while controlling for sex and education level as nuisance variables (corrected *P*_alpha_ of <.05 by Monte Carlo simulation). The interaction remains significant (*P* = .019) after removing outlier subjects with extreme putamen volume on their age.
